# Investigating the renoprotective effects of *Polygonatum sibiricum* polysaccharides (PSP) on D-galactose-induced aging mice: insights from gut microbiota and metabolomics analyses

**DOI:** 10.3389/fmicb.2025.1550971

**Published:** 2025-04-15

**Authors:** Rui Huang, Runli Che, Taoli Sun, Wen Xie, Shuihan Zhang

**Affiliations:** ^1^Hunan Academy of Chinese Medicine, Hunan University of Chinese Medicine, Changsha, China; ^2^The First People's Hospital of Changde City, Changde, China

**Keywords:** *Polygonatum sibiricum* polysaccharides, renoprotection, aging, gut microbiota, metabolomics

## Abstract

**Introduction:**

Polygonatum sibiricum polysaccharides (PSP) have been suggested to possess various health benefits, including anti-aging and renoprotective effects. However, the mechanisms underlying PSP’s protective effects on kidney function, particularly in the context of aging, remain unclear. This study explores how PSP protects against D-galactose (D-gal)-induced kidney damage in aging mice, focusing on gut microbiota and metabolomics.

**Methods:**

Mice were assigned to five groups: control, model (D-gal), vitamin C, low-dose PSP, and high-dose PSP, and treated for 8 weeks. Kidney pathology was assessed via H&E and Masson’s trichrome staining. 16S rRNA sequencing analyzed gut microbiota, and non-targeted metabolomics identified metabolic changes. Correlations between gut bacteria and metabolites were examined.

**Results:**

PSP alleviated renal damage, reducing tubular atrophy, epithelial swelling, and collagen deposition. It increased beneficial gut bacteria (e.g., Lactobacillus, Bifidobacterium) and altered 23 metabolites linked to pathways such as amino acid and sphingolipid metabolism. Gut microbiota and metabolites were strongly correlated, indicating PSP’s role in regulating the gut-kidney axis.

**Conclusion:**

PSP protects against age-related kidney damage by modulating gut microbiota and metabolic pathways, highlighting its therapeutic potential for kidney aging through the gut-kidney axis.

## 1 Introduction

Life expectancy continues to rise, leading to a significant expansion of the elderly population worldwide. This accelerated aging phenomenon presents major challenges to global healthcare systems, as the increasing prevalence of age-related diseases has profound implications for public health, economics, and society. Aging is characterized by complex biological processes involving molecular, structural, and physiological alterations across multiple organs, particularly the kidneys. These age-related changes manifest as metabolic disorders, declining cognitive function and memory, compromised immunity, and increased susceptibility to various diseases and mortality (Bao et al., 2023; Guo et al., 2022; Li et al., 2023). The kidney, a vital organ that maintains homeostasis through waste excretion and blood filtration, undergoes progressive functional decline and structural changes during aging. These age-associated alterations manifest as glomerulosclerosis, tubular atrophy, epithelial cell swelling, atherosclerosis, and interstitial fibrosis. Such changes compromise the kidney’s ability to perform its essential functions, increasing susceptibility to chronic kidney disease and heightening the risk of acute kidney injury (Nossa et al., 2010; Rex et al., 2023). Currently, effective therapeutic interventions to delay kidney aging progression remain limited, making age-related renal dysfunction a critical challenge in modern medicine. Emerging evidence highlights the crucial role of gut microbiota in both the pathogenesis of kidney disorders and the host aging process (Chen et al., 2019; Kim and Benayoun, 2020).

Gut microbiota dysbiosis not only compromises host gastrointestinal function but also accelerates the progression of various kidney disorders, resulting in structural and functional alterations in the renal system (Giordano et al., 2021; Nallu et al., 2017). The mechanisms connecting gut microbiota and kidney disease establish a complex bidirectional relationship, known as the gut-kidney axis (Lan et al., 2023).

*Polygonati Rhizoma*, derived from the dried rhizomes of *Polygonatum kingianum*, *Polygonatum sibiricum*, and *Polygonatum cyrtonema*, has been used in traditional Chinese medicine to tonify qi, nourish yin, and enhance kidney function. Its main bioactive components, *Polygonatum sibiricum* polysaccharides (PSP) are primarily composed of nine monosaccharides: glucose, xylose, mannose, galactose, fructose, rhamnose, arabinose, galacturonic acid, and glucuronic acid (Zhao et al., 2018). Pharmacological studies have demonstrated that PSP possesses diverse biological activities, including antioxidant, anti-fatigue, anti-aging, anti-inflammatory, hypoglycemic, and hypolipidemic effects (Gong et al., 2023; Liu et al., 2022). Studies indicate that PSP can attenuate D-gal-induced kidney aging, enhance renal function, and improve cognitive performance, particularly learning and memory capabilities (Zheng, 2020). Metabolomics studies suggest that PSP protects against renal aging by regulating multiple metabolic pathways, including purine, sphingolipid, glycerophospholipid, tryptophan, and riboflavin metabolism (Zhao et al., 2023). However, the precise mechanisms through which PSP ameliorates kidney aging and modulates gut microbiota composition remain unclear. While multi-omics approaches in microbiome research have provided new insights into complex host–microbe interactions (Shen et al., 2023).

Our previous research explored PSP’s renoprotective mechanisms and their relationship with metabolic regulation through metabolomics analysis. The current study investigates the role of gut microbiota in D-galactose-induced aging mice and PSP’s protective mechanisms using an integrated approach of metabolomics and 16S rRNA sequencing. This methodology enables evaluation of PSP’s effects on renal dysfunction through the gut-kidney axis, offering insights for potential therapeutic applications of *Polygonati Rhizoma* in age-related kidney disorders.

## 2 Materials and methods

### 2.1 Chemicals and reagents

Jiu Huang Jing was procured from Guangdong Huiqun Chinese Medicine Slice Co. The following reagents and materials were obtained from their respective suppliers: purified water (China Resources C’estbon Beverage Co., Ltd.), anhydrous ethanol (China National Pharmaceutical Group Chemical Reagent Co., Ltd.), D-galactose, and ascorbic acid (Sigma, USA), H&E and Masson staining kits (Wuhan Seville Technology Co., Ltd.), and DNA extraction kits (Beijing BioTeke Corporation). Additionally, methanol (Supelco Co., Ltd., USA), formic acid (Tedia, USA), and L-2-chlorophenylalanine (Energy Chemical Reagents, China) were also acquired for the study.

### 2.2 Preparation of PSP

An appropriate amount of dried Jiu Huang Jing herbs was ground into a fine powder at 60°C. The polysaccharides were extracted using a combination of water extraction and ethanol precipitation techniques (Liu et al., 2021). Initially, the medicinal materials were subjected to reflux extraction at 80°C for 1 h, with two repetitions. The first extraction was performed using 10 times the volume of distilled water, while the second extraction utilized 8 times the volume. The resulting filtrate was concentrated under reduced pressure until it reached a specific volume. Subsequently, ethanol precipitation was carried out by adding 4 times the volume of ethanol to the concentrated filtrate, followed by centrifugation at 4°C for 24 h (10,000 rpm, 10 min). The precipitated solid substance was collected, dissolved in a small amount of water, and then freeze-dried to obtain crude polysaccharides. The extracted polysaccharides were stored in a dry place for further use.

### 2.3 Determination of PSP content

The anthrone-sulfuric acid method was used to determine the PSP content. Briefly, 0.25 g of the dried and powdered sample was extracted with 80% ethanol and water successively under reflux. The combined filtrate and washing solution were made up to 250 mL. A 1 mL aliquot of the sample solution was mixed with water and 0.2% anthrone-sulfuric acid solution in a test tube. The mixture was heated at 100°C for 10 min, cooled in an ice-water bath, and the absorbance was measured at 582 nm. After linear regression, the standard curve obtained is: y = 44.316x +0.051, R^2^ = 0.9978. In the equation, y represents the absorbance, and x represents the concentration of the standard substance.


$$PSP\left(\%\right)=\frac{C\times V\times D}{1000\times W0}\times 100\%$$

In the formula: C, concentration of the test sample (mg/mL), calculated from the standard curve; V, volume of the test sample (mL); D, dilution factor; W, weight of the test sample (g). The PSP content in *Polygonatum sibiricum*, as determined by the anthrone-sulfuric acid method, was found to be 12.82%.

### 2.4 Animals and experimental design

Male C57BL/6 J mice (Hunan Slack Jingda Experimental Animal Co., Ltd.) were acclimated for 1 week before experiments. Animals were housed at 25 ± 2°C with a 12-h light/dark cycle and free access to food and water. All procedures followed guidelines approved by the Ethical Committee for Animal Experiments at Hunan Academy of Chinese Medicine.

Thirty mice were randomly divided into five groups (*n* = 6 per group): Control group (CON), Model control group (MOD), Vitamin C group (VC), Low-dose PSP group (LPSP, 150 mg/kg/d), High-dose PSP group (HPSP, 600 mg/kg/d). To induce aging, all groups except CON received intraperitoneal injections of D-gal (150 mg/kg/d in normal saline). The CON group received equivalent volumes of normal saline. Treatment administration was as follows: LPSP and HPSP groups intragastric gavage with 150 mg/kg/d PSP and 600 mg/kg/d PSP, respectively; VC group intragastric gavage with 300 mg/kg/d VC; CON and MOD groups intragastric gavage with distilled water. The experiment lasted 8 weeks, with oral treatments beginning at week five.

### 2.5 Sample collection and processing

After the final administration, mice were fasted for 24 h. Blood was collected via retro-orbital bleeding under 1% pentobarbital sodium anesthesia. Serum was obtained by allowing blood samples to clot for 1 h, followed by centrifugation at 3000 rpm for 10 min at 4°C. Kidneys were excised, weighed, and rinsed with cold saline. For histopathological analysis, kidney portions were fixed in 4% paraformaldehyde, embedded in paraffin, and sectioned for H&E and Masson staining. The remaining kidney tissue and fecal samples were flash-frozen in liquid nitrogen and stored at −80°C for subsequent analyses.

### 2.6 Histopathological assessment

For histological analysis, kidney tissues were fixed in 4% paraformaldehyde, dehydrated, embedded in paraffin, and sectioned to 5 μm thickness. Sections were stained with H&E and Masson techniques. Morphological and pathological changes were assessed by light microscopy and documented photographically.

### 2.7 Gut microbiota analysis by 16S rDNA sequencing

Fecal DNA was extracted using the MagPure Soil DNA LQ kit (Magan). DNA quality and purity were assessed via NanoDrop 2000 spectrophotometer (Thermo Fisher Scientific, USA) and agarose gel electrophoresis. Samples were stored at −20°C until analysis. The 16S rRNA gene amplification was performed using Takara Ex Taq polymerase with barcoded primers: Forward (343F): 5’-TACGGRAGG CAGCAG-3′; Reverse (798R): 5’-AGGGTATCTA ATCCT-3′. PCR products were purified using AMPure XP beads, quantified with Qubit dsDNA HS Assay Kit (Thermo Fisher Scientific, USA), and sequenced on the Illumina NovaSeq 6000 platform to generate 250 bp paired-end reads.

### 2.8 HPLC-QE-MS/MS for non-targeted metabolomics analysis

An UltiMate 3,000 UHPLC system coupled with a Thermo Q-Exactive Orbitrap mass spectrometer equipped with electrospray ionization (ESI) was employed to analyze fecal and serum metabolites. The mass spectrometry parameters were set as follows: (1) data quality scan range from 60 to 900 Da; (2) full scan mass resolution of 70,000; (3) MS2 resolution of 17,500; (4) sheath gas flow rate of 30 Arb and auxiliary gas flow rate of 10 Arb; (5) capillary temperature of 320°C and auxiliary gas heating temperature of 375°C; (6) positive ion mode at 3500 V and negative ion mode at 3100 V.

Metabolite separation was achieved using an ACQUITY UPLC HSS T3 column (100 mm × 2.1 mm, 1.8 μm) (Waters Corporation, Milford, MA, United States) with a gradient elution of 0.1% formic acid in water (phase A) and methanol (phase B). The flow rate was set at 0.35 mL/min, column temperature at 45°C, and injection volume at 10 μL. The gradient elution was programmed as follows: 0–1.5 min, 95% A; 1.5–3 min, 95–70% A; 3–5 min, 70–40% A; 5–7 min, 40–20% A; 7–12 min, 20–0% A; 12–16 min, 0% A; 16–16.5 min, 0–95% A.

### 2.9 Data processing and analysis

The raw data collected using Xcalibur 4.2 SP1 software underwent format conversion, peak extraction, peak filtering, and peak matching. Subsequently, the data was calibrated based on quality control (QC) samples. The processed data was then matched with mass spectrometry (MS) information to obtain a final data matrix. OSI/SMMS software, developed by the Dalian Institute of Chemical Physics, Chinese Academy of Sciences, and Dalian Chem Data Solution Information Technology Co., Ltd., was employed for identifying differential metabolites. The analysis was performed using the One-Map metabolomics data analysis cloud platform,^1^ and SIMCA 18.0 was used for data visualization. Differential metabolites were further screened and identified based on a variable importance in projection (VIP) score > 1 and a *p*-value <0.05, using the Human Metabolome Database (HMDB)^2^ and PubChem^3^ as reference databases. The identified differential metabolites were then subjected to pathway enrichment analysis using the MetaboAnalyst 6.0 platform, which is freely available online.

### 2.10 Correlation analysis

The relationships between differential microbial genera and altered metabolites were assessed using Spearman correlation analysis, integrating data from 16S rRNA sequencing and metabolomic profiling of fecal and serum samples.

### 2.11 Statistical analysis

Statistical analyses and data visualization were performed using SPSS 25.0 and GraphPad Prism 8.0 software. The data are presented as mean ± standard deviation (SD). Analysis of variance (ANOVA) was used for data that were normally distributed with equal variances; otherwise, a non-parametric test was employed. A *p*-value <0.05 was considered statistically significant.

## 3 Results

### 3.1 PSP alleviates kidney pathological changes induced by D-gal in aging mice

**Figure 1:**
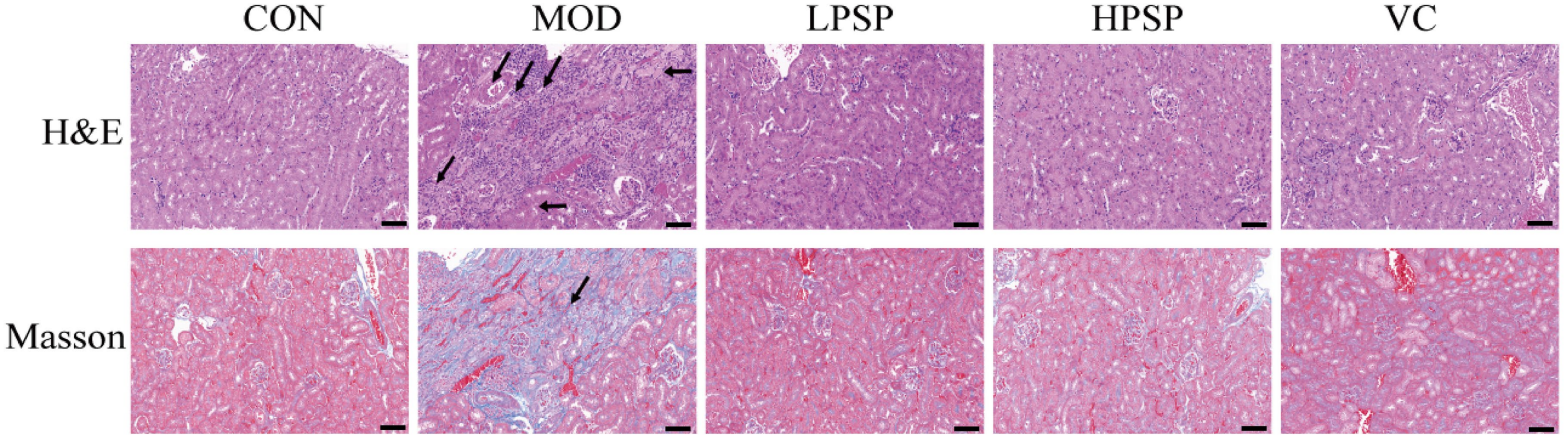
Pathological changes of kidney tissue (200X, scale bar = 200 μm).

The presents H&E and Masson staining results were shown in Figure 1. Compared to CON group, MOD group exhibited renal damage characterized by focal tubular atrophy, reduced tubular volume, epithelial cell edema, inflammatory cell infiltration, and increased interstitial collagen deposition (black-stained fibers). Four weeks of PSP treatment attenuated these pathological changes, aligning with previous findings (Pan et al., 2021). The findings suggest that PSP can mitigate kidney pathological damage and reduce fibrosis. Based on earlier research (Zhao et al., 2023), HPSP was selected as the representative treatment group for further analysis.

### 3.2 The impact of PSP on the intestinal microbiota in D-gal-induced mice

#### 3.2.1 Diversity analysis of gut microbiota

**Figure 2:**
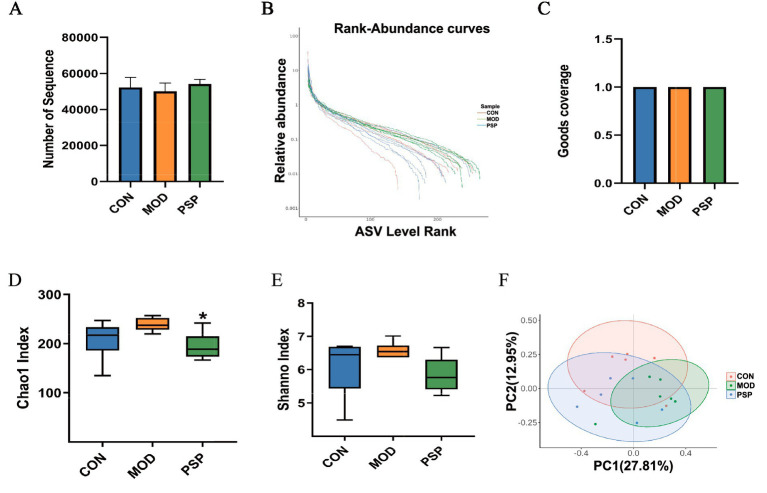
Diversity analysis of gut microbiota in mice (*n* = 6). **(A)** Number of Sequences. **(B)** Rank abundance distribution curve. **(C)** Good_coverage. **(D)** Chao1 index. **(E)** Shannon index. **(F)** PCoA analysis. Data are mean ± SD, ^*^p < 0.05 (MOD vs. PSP).

The analysis of the initial sequencing data yielded a total of 938,687 sequences (Figure 2A). A rank abundance distribution curve was then plotted to assess the diversity and uniformity of the intestinal microbiota across groups (Figure 2B). The Good’s coverage for each sample approached 1 (Figure 2C), indicating that the sequencing results accurately reflect the true conditions of the samples.

Gut microbial richness and evenness were assessed using Chao1 and Shannon indices. MOD group showed elevated Chao1 and Shannon indices compared to CON group. PSP treatment restored the Chao1 index (*p* < 0.05) and partially normalized the Simpson index (Figures 2D,E). These results suggest D-gal-induced alterations in intestinal microbial diversity were ameliorated by PSP.

Furthermore, principal coordinate analysis (PCoA) using Bray-Curtis distance showed a clear separation in microbial compositions between the CON and MOD groups, indicating significant shifts in gut microbiota due to D-gal-induced aging (Figure 2F).

#### 3.2.2 Alterations in gut microbiota composition

**Figure 3:**
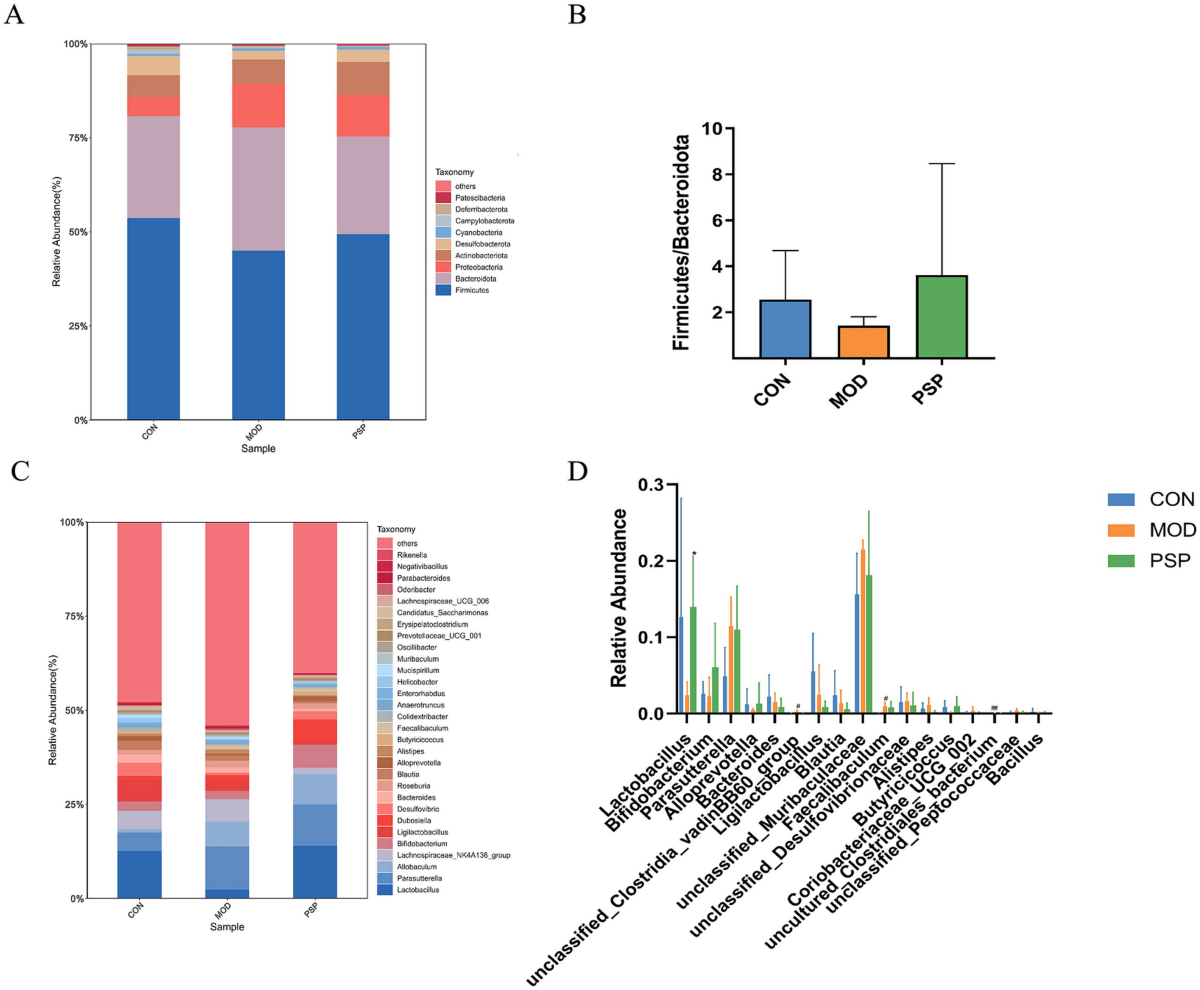
Alterations in gut microbiota composition at the phylum and genus levels in each group of mice. **(A,C)** Phylum and genus level. **(B)** Ratio of Firmicutes to Bacteroidetes. **(D)** Significant changes in abundance at the genus levels. Data are mean ± SD, ^*^p < 0.05, ^**^p < 0.01 (MOD vs. PSP); ^#^*p* < 0.05, ^##^*p* < 0.01 (CON vs. MOD).

To better understand how PSP regulates the gut microbiota in mice, we analyzed the composition of the intestinal microbiota at various taxonomic levels. The results indicated that the dominant phyla in the mouse gut microbiota were primarily Firmicutes, Bacteroidota, Proteobacteria, and Actinobacteria (Figure 3A). Firmicutes and Bacteroidota were the predominant phyla, reflecting the overall gut microbiota profile.

In the MOD group, there was a decrease in the relative abundance of Firmicutes and an increase in Bacteroidota. However, PSP intervention attenuated this decline in Firmicutes and the rise in Bacteroidota. The Firmicutes/Bacteroidota ratio (F/B) is commonly used to assess gut microbiota imbalance. In the model group, the F/B ratio decreased compared to the normal group, but PSP treatment partially restored this ratio, suggesting a trend toward balancing the gut microbiota composition, as illustrated in Figure 3B. Although these changes did not reach statistical significance, they indicate PSP’s potential role in regulating gut microbiota alterations in model mice.

At the genus level, the mouse gut microbiota was predominantly composed of *Parasutterella*, *Lactobacillus*, *Allobaculum*, *Lachnospiraceae*_NK4A136_group, and *Bifidobacterium* (Figure 3C). D-gal treatment resulted in a decline in the populations of *Lactobacillus*, *Bifidobacterium*, and *Alloprevotella*, among others. In contrast, PSP treatment led to an increase in the abundance of these beneficial genera (Figure 3D).

We employed the LEfSe analytic method to analyze various species across multiple hierarchical levels and identify the microorganisms with relatively elevated abundance in each group, using a default LDA value of 2. The analysis of the CON and MOD groups produced histograms, cladograms, and comparisons of significant differences in biomarker abundance among the groups.

This analysis revealed that 21 taxa were identified at different taxonomic levels. Of these, 10 taxa were found in the model group, including dominant bacteria such as *Erysipelotrichales*, *Allobaculum*, *Clostridia*_vadinBB60_group, *Proteobacteria*, and *Parasutterella*. The remaining 11 taxa were present in the normal group, primarily comprising *Lactobacillales*, *Campylobacterota*, *Streptococcaceae*, and *Butyricicoccaceae* (Figures 3C,D).

#### 3.2.3 LEfSe analysis of dominant biomarker taxa

**Figure 4:**
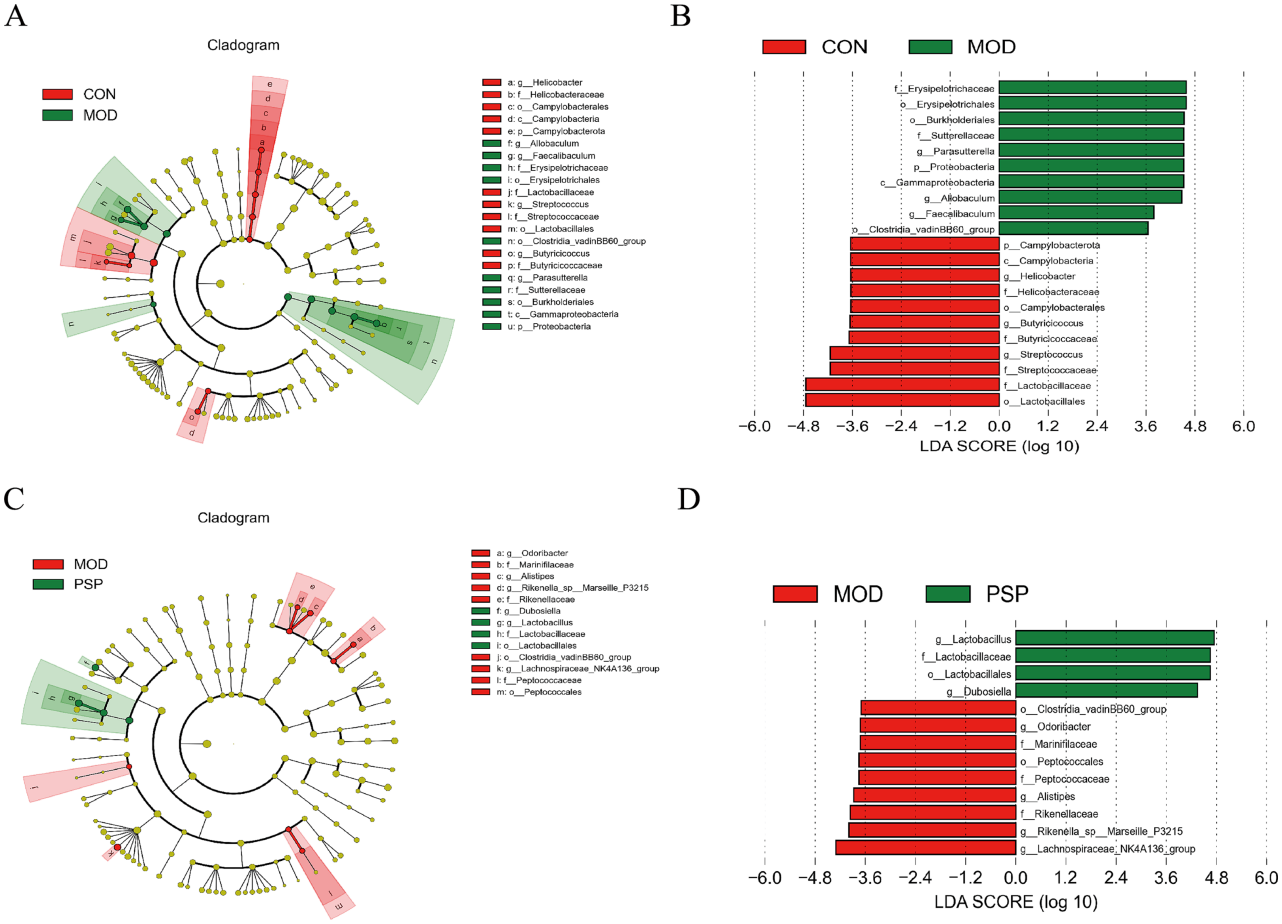
LEfSe analysis of dominant biomarker taxa among the three groups. **(A,C)** Cladograms illustrating the microbial communities that play significant roles in the comparisons of CON vs. MOD and MOD vs. PSP. **(B,D)** The LEfSe method identifies statistically significant biomarkers between the CON and MOD groups, as well as between the MOD and PSP groups.

Compared to the control group (CON), 21 distinct bacterial taxa were identified at various taxonomic levels (Figure 4A). In the PSP-treated group versus the model group (MOD), we identified 13 differentially abundant taxa: *Odoribacter*, *Marinifilaceae*, *Alistipes*, *Rikenella*_sp_Marseille_P3215, *Rikenellaceae*, *Dubosiella*, *Lactobacillus*, *Lactobacillaceae*, *Lactobacillales*, *Clostridia*_vadinBB60_group, *Lachnospiraceae*_NK4A136_group, *Peptococcaceae*, and *Peptococcales* (Figure 4B). Of these taxa, 9 showed significant changes in the model group, while 4 were specifically altered in the PSP treatment group (Figure 4C). Notably, *Lactobacillales* emerged as the predominant taxonomic group in response to PSP treatment (Figure 4D).

### 3.3 Effects of PSP intervention on serum and fecal metabolites in aging mice

#### 3.3.1 Multivariate statistical analysis

**Figure 5:**
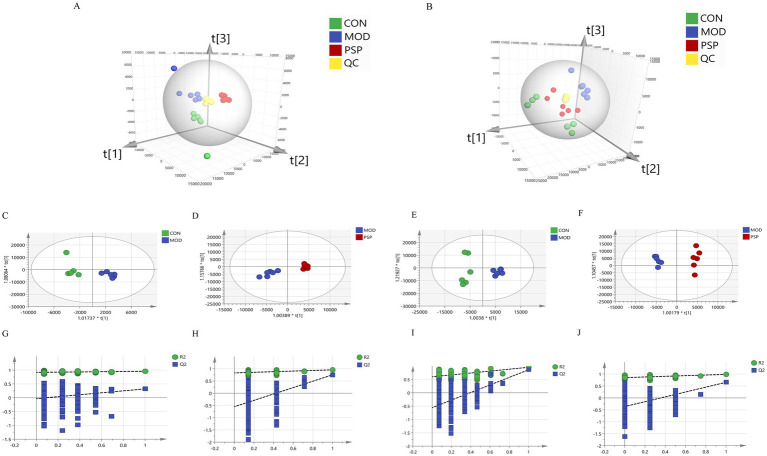
Multivariate statistical analysis results. **(A)** Serum PLS-DA; **(B)** Feces PLS-DA; **(C,E)** OPLS-DA plots of serum and feces for CON and MOD groups; **(D,F)** OPLS-DA plots of serum and feces for MOD and PSP groups; **(G,H)** 200 permutation tests for CON and MOD groups; **(I,J)** 200 permutation tests for MOD and PSP groups.

Partial Least Squares Discriminant Analysis (PLS-DA) was employed to analyze overall discrepancies in serum and fecal metabolites, revealing significant distinctions among the groups. Notably, the PSP group exhibited a profile closely resembling that of the CON group, as shown in Figures 5A,B. The OPLS-DA score plots clearly demonstrate distinct clustering and separation of metabolites among the different groups in various regions (Figures 5C–F).

Additionally, we assessed the parameters of the OPLS-DA model (Figures 5G–J), where R^2^Y indicates the percentage of variation in the Y matrix explained by the model, and Q^2^ reflects the model’s predictive capability. Higher values of R^2^Y and Q^2^ approaching 1 indicate enhanced stability and reliability, suggesting that the sample model is robust and possesses strong predictive ability. For the CON and MOD groups, the values were R^2^Y(cum) = 0.955 and Q^2^(cum) = 0.861, and for the MOD and PSP groups, the values were R^2^Y(cum) = 0.986 and Q^2^(cum) = 0.653. These results affirm the reliability of the model.

#### 3.3.2 Analysis of differential metabolites and pathway enrichment

**Figure 6:**
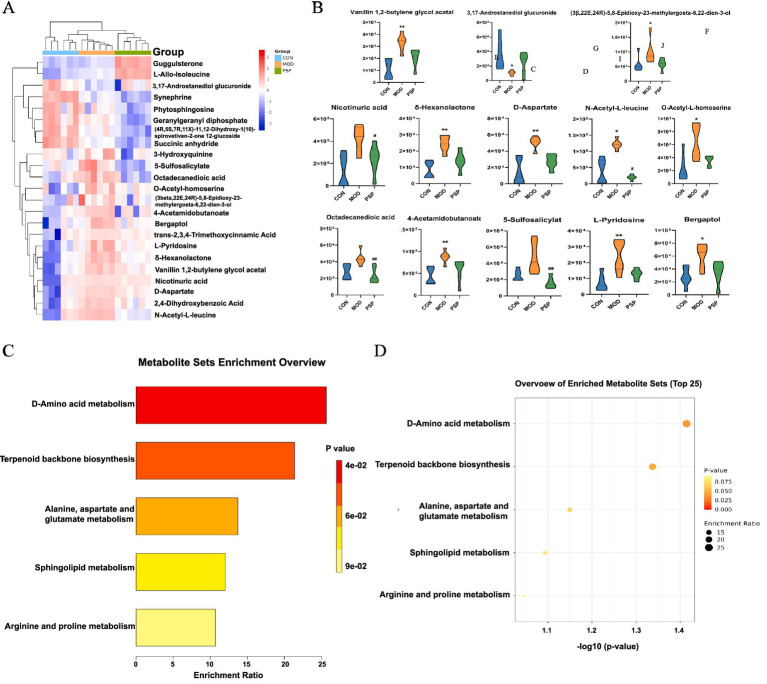
Differential metabolite and pathway enrichment analysis. **(A)** Hierarchical clustering heatmap. **(B)** Changes in potential biomarkers. **(C,D)** KEGG pathway enrichment analysis.

Using a combined approach with the HMDB and PubChem databases, a total of 23 metabolites were identified as significantly altered between the control (CON), model (MOD), and PSP-treated groups. These metabolites were primarily involved in amino acid metabolism (e.g., D-aspartate, L-alanine), lipid metabolism (e.g., phytosphingosine, octadecanedioic acid), and other pathways such as sphingolipid metabolism, as detailed in Table 1. Among these, 12 metabolites were significantly upregulated and 11 were significantly downregulated compared to the CON group. Notably, the expression levels of these metabolites showed a substantial reversal following PSP treatment (Figures 6A,B).

**Table 1:** Differential metabolites in serum and feces.

NO.	Metabolites	Formula	m/z	HMDB	Variations	
					CON vs. MOD	MOD vs. PSP
SM1	5-Sulfosalicylate	C_7_H_6_O_6_S	218.994958	HMDB0011725	↓^*^	↑^##^
SM2	3-Hydroxyquinine	C_20_H_24_N_2_O_3_	341.1786736	HMDB0001091	↓^*^	↑^#^
SM3	Geranylgeranyl diphosphate	C_20_H_36_O_7_P_2_	451.1927126	HMDB0004486	↓^*^	↑^###^
SM4	Guggulsterone	C_21_H_28_O_2_	625.4198466	HMDB0002726	↓^*^	↑^###^
SM5	O-Acetyl-L-homoserine	C_6_H_11_NO_4_	160.0607021	HMDB0029423	↑^**^	↓^#^
SM6	Octadecanedioic acid	C_18_H_34_O_4_	313.2383881	HMDB0000782	↓^**^	↑^##^
SM7	(4R,5S,7R,11x)-11,12-Dihydroxy-1 (10)-spirovetiven-2-one 12-glucoside	C_21_H_34_O_8_	413.2181966	HMDB0030895	↓^**^	↑^##^
FM1	Succinic anhydride	C_4_H_4_O_3_	101.0211282	HMDB0032523	↓^***^	↑^#^
FM2	δ-Hexanolactone	C_6_H_10_O_2_	115.0754681	HMDB0000453	↑^***^	↓^##^
FM3	Synephrine	C_9_H_13_NO_2_	150.091343	HMDB0004826	↓^***^	↑^##^
FM4	2,4-Dihydroxybenzoic Acid	C_7_H_6_O_4_	155.0313875	HMDB0029666	↑^**^	↓^##^
FM5	Nicotinuric acid	C_8_H_8_N_2_O_3_	181.0607473	HMDB0003269	↑^**^	↓^#^
FM6	Bergaptol	C_11_H_6_O_4_	203.0424329	HMDB0013679	↑^*^	↓^#^
FM7	Vanillin 1,2-butylene glycol acetal	C_12_H_16_O_4_	225.1097089	HMDB0032552	↑^***^	↓^##^
FM8	L-Pyridosine	C_12_H_18_N_2_O_4_	255.1334255	HMDB0029443	↑^**^	↓^#^
FM9	Phytosphingosine	C_18_H_39_NO_3_	318.2997231	HMDB0004610	↓^*^	↑^##^
FM10	(3β,22E,24R)-5,8-Epidioxy-23-methylergosta-6,22-dien-3-ol	C_29_H_4_6O_3_	443.3518107	HMDB0032668	↑^*^	↓^#^
FM11	L-Allo-Isoleucine	C_6_H_13_NO_2_	130.0863696	HMDB0000557	↑^**^	↓^##^
FM12	D-Aspartate	C_4_H_7_NO_4_	132.0373437	HMDB0006483	↑^**^	↓^##^
FM13	4-Acetamidobutanoate	C_6_H_11_NO_3_	144.0657366	HMDB0003681	↓^**^	↓^#^
FM14	N-Acetyl-L-leucine	C_8_H_15_NO_3_	172.09719	HMDB0011756	↑^**^	↓^#^
FM15	trans-2,3,4-Trimethoxycinnamic Acid	C_12_H_14_O_5_	237.0801134	HMDB0011721	↑^*^	↓^#^
FM16	3,17-Androstanediol glucuronide	C_25_H_40_O_8_	467.2653554	HMDB0010321	↓^**^	↑^##^

SM and FM represent serum metabolites and fecal metabolites respectively, “↑” Representative up-regulation, “↓” Representative down-regulation. Compared with CON, ^*^p < 0.05, ^**^p < 0.01, ^***^p < 0.001; Compared with MOD, ^#^*p* < 0.05, ^##^*p* < 0.01, ^###^*p* < 0.001.

Enrichment analysis indicated that PSP treatment significantly impacted pathways including D-amino acid metabolism, alanine, aspartate and glutamate metabolism, terpenoid backbone biosynthesis, and sphingolipid metabolism. These pathways are associated with mitochondrial function, oxidative stress, and lipid peroxidation, all of which play critical roles in aging and renal dysfunction (Figures 6C,D).

### 3.4 Correlation analysis of gut microbiota with serum and fecal metabolomics

We conducted Spearman correlation analysis to investigate the interactions between 23 endogenous differential metabolites and the top 15 gut microbiota genera at the genus level, aiming to explore the relationship between gut bacteria and host metabolism. Our findings revealed that specific metabolites exhibited significant correlations with distinct gut flora.

Notably, the gut microbiota primarily influenced variations in metabolites associated with amino acids and lipids. For instance, *Ligilactobacillus* showed a positive correlation with phytosphingosine and succinic anhydride, while *Allobaculum* was positively associated with D-aspartate, O-acetyl-L-homoserine, (3B,22E,24R)-5,8-epidioxy-23-methylergosta-6,22-dien-3-one, and L-allo-isoleucine. Additionally, *Ligilactobacillus* was positively correlated with both nicotinuric acid and L-allo-isoleucine.

**Figure 7:**
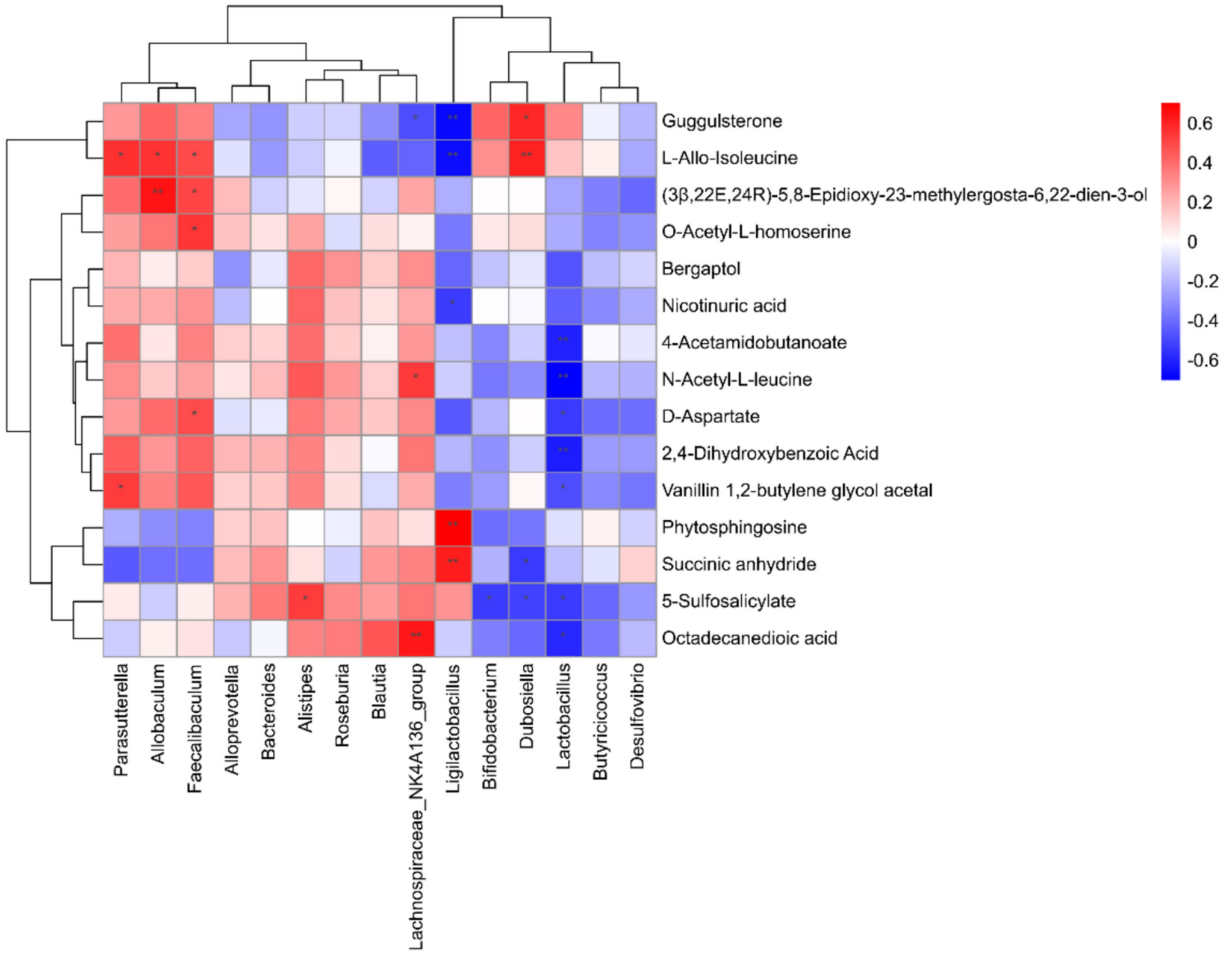
Analysis of the correlation between gut microbiota and differential metabolites.

Conversely, *Lactobacillus* demonstrated a negative correlation with octadecanedioic acid, 5-sulfosalicylic acid, vanillin 1,2-butylene glycol acetal, 2,4-dihydroxybenzoic acid, D-aspartate, N-acetyl-L-leucine, and 4-acetamidobutanoate. Furthermore, *Bifidobacterium* also exhibited a negative correlation with 5-sulfosalicylic acid, as illustrated in Figure 7.

## 4 Discussion

According to “*Su Wen: Shang Gu Tian Zhen Lun*,” kidney qi plays a crucial role in human growth, development, and aging. Academician Wu Yiling proposed that kidney essence deficiency and diminished vitality are key factors in aging, manifested as a decline in both physical and mental vigor. Based on this understanding, Wu developed a therapeutic strategy emphasizing kidney nourishment, essence replenishment, and qi restoration to maintain both physical and spiritual well-being (Guo et al., 2021). *Polygonatum sibiricum*, a neutral-natured herb with sweet taste, acts on spleen, lung, and kidney meridians. It functions to nourish qi and yin, strengthen spleen, moisten lungs, and tonify kidneys (Chinese Pharmacopoeia Commission, 2020). Its polysaccharides demonstrate anti-aging effects through metabolic regulation, establishing its importance in traditional Chinese medicine aging interventions.

Our previous research established PSP’s capacity to regulate renal aging biomarkers, including adenosine monophosphate (AMP), tryptophan, and 5-hydroxytryptophan. To expand upon these findings, we investigated PSP’s gut-kidney axis mechanisms using a D-gal-induced aging model. Through comprehensive microbiome and metabolome analyses, we demonstrated that PSP mitigates age-related renal pathology by normalizing gut microbiota composition and associated metabolic profiles.

Aging reduces microbial diversity, marked by decreased *Bifidobacterium* and *Lactobacillus* populations alongside increased *Firmicutes* and *Proteobacteria* (Biagi et al., 2016; Milenkovic et al., 2023; Xie et al., 2020). Our 16S rRNA sequencing revealed disrupted gut microenvironment in aging mice, characterized by altered abundances of *Firmicutes*, *Bacteroidetes*, *Actinobacteria*, and *Proteobacteria*. This dysbiosis compromises intestinal barrier integrity, enabling bacterial translocation and promoting chronic low-grade inflammation. Aging-related changes at the genus level include decreased beneficial bacteria, notably *Lactobacillus* and *Bifidobacterium*. This study demonstrated that PSP exerts renoprotective effects in D-gal-induced aging mice by modulating the gut-kidney axis. The treatment alleviated renal pathological changes, restored gut microbiota balance, and regulated key metabolic pathways. These findings are consistent with the studies by Hsiao et al. (2021), which highlight the role of probiotics, such as *Lactobacillus* and *Bifidobacterium*, in attenuating cisplatin-induced renal damage through gut microbiota reconstitution, increased butyric acid production, and suppression of renal inflammation. PSP’s ability to increase these beneficial genera supports its potential as a gut microbiota modulator. *Bifidobacterium* contributes to alanine and aspartic acid production. D-aspartate shows negative correlation with *Lactobacillus*, while nicotinuric acid correlates with *Ligilactobacillus*. Lactic acid-producing probiotics preserve renal function through intestinal barrier repair, inflammation reduction, and oxidative stress attenuation (Yoshifuji et al., 2016). *Bifidobacterium* enhances intestinal immunity and inversely correlates with chronic inflammation in elderly populations, protecting kidneys by inhibiting macrophage activation and inflammatory responses (Kim et al., 2023; Rui-Zhi et al., 2020). Modulation of short-chain fatty acids (SCFAs) attenuates kidney damage and functional decline. *Alloprevotella*, associated with butyric and acetic acids, produces lactate as a substrate for butyrate-producing bacteria (Brame et al., 2021; Zhu et al., 2021). SCFA-producing probiotics mediate PSP’s effects on gut microbiota, potentially ameliorating renal aging through the gut-kidney axis. PSP appears to increase beneficial bacterial populations, promoting renoprotection.

Correlation analysis between gut microbiota and serum/fecal metabolomics revealed that intestinal flora primarily regulates differential metabolites in amino acids and lipids. *Ligilactobacillus* showed positive correlations with phytosphingosine, succinic anhydride, and *Bacteroides* correlated positively with D-aspartic acid, O-acetyl-L-homoserine, and L-alanine. *Lactobacillus* negatively correlated with Octadecanedioic acid, 5-sulfosalicylic acid, vanillin-1,2-butanediol acetal, 2,4-dihydroxybenzoic acid, D-aspartic acid, N-acetyl-L-leucine, and 4-acetylaminobutyric acid. *Bifidobacterium* displayed negative correlation with 5-sulfosalicylate. Studies have demonstrated that D-aspartic acid and L-alanine participates in lipid peroxidation processes (Chandrashekar and Muralidhara., 2010; Parildar et al., 2008). Therefore, *Bacteroides* regulation of differential lipid metabolites may be associated with lipid peroxidation.

Previous research by Zhao et al. (2023) demonstrated that PSP regulates pathways such as purine and sphingolipid metabolism. Our study not only corroborates these findings but also expands on them by identifying additional pathways, including D-amino acid metabolism and lipid peroxidation, as critical mechanisms involved in renal aging. Lipid peroxidation, a process enhanced by intestinal fatty acids such as D-aspartic acid and L-alanine, plays a crucial role in promoting ferroptosis (Chandrashekar and Muralidhara., 2010; Giacone et al., 2017). This iron-dependent cell death occurs when accumulated iron generates reactive oxygen species (ROS) through Fenton reactions, further accelerating the oxidation of membrane polyunsaturated fatty acids. When antioxidant systems fail to counter this oxidative cascade, the resulting lipid peroxide accumulation causes membrane damage and cell death through a pathway distinct from apoptosis or necrosis (Jiang et al., 2021). Recent studies have demonstrated that PSP can protect against D-gal-induced cognitive dysfunction in aging rats by activating Nrf2, a key antioxidant regulator that inhibits this lipid peroxidation-driven ferroptotic process (Xiao et al., 2024). Jiang et al. (2021) highlighted the role of lipid peroxidation in ferroptosis, a process that contributes to renal aging. Our study provides evidence that PSP mitigates ferroptosis by reducing lipid peroxidation. These findings align with those of Xiao et al. (2024), who demonstrated the antioxidant effects of PSP in aging models. Therefore, we propose that PSP’s renoprotective effects against D-gal-induced aging through the gut-kidney axis are also associated with ferroptosis inhibition.

The main limitation of this study is the absence of comprehensive experimental validation of PSP’s anti-renal aging effects through ferroptosis inhibition at both cellular and animal levels. Future research should address this gap through integrated *in vitro* and *in vivo* studies to definitively establish ferroptosis inhibition as a key mechanism underlying renoprotective effects of PSP during aging. Additionally, investigating other potential anti-aging mechanisms will provide a more complete understanding of PSP’s therapeutic potential in age-related kidney disorders.

## 5 Conclusion

Using D-gal-induced aging models, we demonstrated that PSP exerts renoprotective effects through modulation of the gut-kidney axis. Our integrated analysis of 16S rRNA sequencing and untargeted metabolomics revealed that aging-induced renal pathological changes were accompanied by gut microbiota dysbiosis and altered metabolic profiles. Notably, PSP treatment normalized gut microbiota composition and regulated key metabolites, particularly those involved in lipid peroxidation pathways. The correlation between gut microbiota and metabolomics showed that intestinal fatty acids participate in lipid peroxidation, which drives ferroptosis. These findings not only enhance our understanding of PSP’s anti-aging mechanisms via the gut-kidney axis but also provide a scientific foundation for its therapeutic development in age-related kidney disorders.

## Data Availability

The original contributions presented in the study are publicly available. This data can be found here: https://doi.org/10.6084/m9.figshare.28736108.

## Footnotes

^1^ http://www.5omics.com/

^2^ https://hmdb.ca/

^3^ https://pubchem.ncbi.nlm.nih.gov/
